# Economic Impact of Severe Early‐Onset Foetal Growth Restriction: A Multicentre Prospective Cohort Study

**DOI:** 10.1111/1471-0528.18266

**Published:** 2025-06-24

**Authors:** George Bray, Kasia Maksym, Maurvi Dilipkumar, Rebecca N. Spencer, Yuval Ginsberg, Tal Weissbach, Donald M. Peebles, Neil Marlow, Angela Huertas‐Ceballos, Gina Buquis, Jade Okell, Kurt Hecher, Anke Diemert, Dominique Singer, Stefan R. Hansson, David Ley, Francesc Figueras, Eduard Gratacós, Fatima Crispi, Anna L. David, Rachael Hunter

**Affiliations:** ^1^ Department of Applied Health Research University College London London UK; ^2^ Elizabeth Garrett Anderson Institute for Women's Health University College London London UK; ^3^ Leeds Institute of Cardiovascular and Metabolic Medicine University of Leeds Leeds UK; ^4^ Department of Obstetrics and Gynecology Rambam Medical Centre Haifa Israel; ^5^ Department of Obstetrics and Gynecology Sheba Tel Hashomer Hospital Tel Aviv Israel; ^6^ Neonatal Department University College London Hospitals NHS Foundation Trust London UK; ^7^ Department of Obstetrics and Fetal Medicine University Medical Center Hamburg‐Eppendorf Hamburg Germany; ^8^ Department of Clinical Sciences Lund, Obstetrics and Gynaecology Skane University Hospital, Lund University Lund Sweden; ^9^ Department of Clinical Sciences Lund, Pediatrics Skane University Hospital, Lund University Lund Sweden; ^10^ BCNatal, Barcelona Center for Maternal‐Fetal and Neonatal Medicine, IDIBAPS, IRSJD, Hospital Clinic, Hospital Sant Joan de Déu, University of Barcelona, CIBERER Barcelona Spain; ^11^ National Institute for Health and Care Research University College London Hospitals Biomedical Research Centre London UK

**Keywords:** economic burden, economic evaluation, foetal growth restriction, healthcare costs, neonatal intensive care, preterm birth

## Abstract

**Objective:**

Foetal growth restriction (FGR) affects 10% of pregnancies, contributing to 30% of stillbirths. Current management of early‐onset FGR (< 32 + 0 weeks' gestation) delivers the foetus before stillbirth or irreversible organ damage. The resulting preterm births create additional risks independent of FGR. We determined the economic cost associated with severe early‐onset FGR.

**Design:**

Economic analysis of EVERREST prospective study, a 6‐year multicentre prospective cohort study.

**Setting:**

UK, Spain, Germany, Sweden.

**Population:**

Pregnant women with estimated foetal weight < 3rd centile, 20 + 0–26 + 6 weeks of gestation.

**Methods:**

Between antenatal recruitment and 2 years post‐delivery, maternal and infant resource use was collected using the Global Pregnancy CoLaboratory (COLAB) data set and an adapted client service receipt inventory (CSRI) questionnaire.

**Main Outcome Measure:**

Cost differences between gestational age groups with Multivariable Generalised Linear Models.

**Results:**

Of 135 births, 46% were extremely preterm (EPT, < 28 + 0 weeks), 23% very preterm (VPT, 28 + 0–< 32 + 0 weeks), 16% late/moderate preterm (MLPT, 32 + 0–< 37 + 0 weeks) and 14% term. Neonatal Unit (NNU) costs accounted for the largest costs incurred by either mother or infant, exhibiting the largest differences between gestational age groups. EPT infants costed an additional £157 832 (95% CI: £96 904–£218 760) on average per infant compared to the term group, VPT infants an additional £93 709 (95% CI: £62 656–£124 761) and MLPT infants an additional £20 182 (£11 882–£28 482).

**Conclusions:**

Early‐onset FGR has substantial costs, predominantly incurred during infants' NNU admissions. Births < 32 + 0 weeks have significantly higher costs than term births, providing economic justification to research therapies that reduce iatrogenic preterm birth.

**Trial Registration:**

ClinicalTrials.gov identifier: NCT02097667

## Abstract 2

**Linked article:** This article is commented on by Sharp, pp. 71 in this issue. To view this article visit https://doi.org/10.1111/1471‐0528.18327.

## 1 Introduction

Foetal Growth Restriction (FGR) is a serious obstetric condition in which foetal growth is impaired. It affects around 10% of all pregnancies and contributes to 30% of stillbirths [1]. In cases of early‐onset FGR which develops before 32 + 0 weeks' gestation, the associated complications arise at a very early stage of pregnancy. FGR is most commonly caused by placental insufficiency with other causes due to maternal diseases such as infections and foetal, chromosomal, genetic or structural anomalies [2]. There is currently no treatment that can improve foetal growth in utero, although a number of interventions have been tested in clinical trials [3]. Instead, current management involves monitoring the pregnancy and timing the birth to balance the risks of stillbirth and prematurity, with delivery often resulting in preterm birth [4]. This creates additional risks due to the complications that result from preterm birth independent of FGR including neonatal morbidity and mortality, neurodevelopmental impairment and long‐term health problems [5, 6, 7].

The EVERREST clinical trial is aiming to assess the safety and efficacy of maternal uterine artery injection of an adenovirus containing the Vascular Endothelial Growth Factor transgene (Ad.VEGF), a gene therapy treatment under development to address FGR caused by placental insufficiency [8]. The greatest potential for benefit is in those most affected pregnancies wherein the foetus is severely small, with an estimated foetal weight (EFW) below the 3rd centile. In such pregnancies, women with experience of FGR and stakeholders considered trialling a novel therapeutic to be ethically and socially acceptable [9].

In preparation for the clinical trial, we established the multicentre, 6‐year EVERREST prospective study across four European centres to characterise the natural history of early‐onset FGR, choosing an extreme ‘severe’ phenotype in which the estimated foetal weight (EFW) was < 3rd centile and < 600 g between 20 + 0 and 26 + 6 weeks of gestation [10]. This paper analyses the economic costs and the predictors of costs within this population. The risks associated with FGR and preterm birth mean there is scope for substantial resource use and costs to occur for both mother and infant, in terms of health service use and broader societal costs such as lost productivity and informal care costs. Establishing the economic burden of severe early onset FGR is important given resources are scarce. Although a cost of illness study will not show how resources are best allocated, it can provide impetus for further research into cost‐effective treatments. There are currently no studies examining the economic burden of severe early onset FGR.

## 2 Methods

### 2.1 Data

#### 2.1.1 Study Population

The EVERREST prospective study recruited participants from University College London Hospital (UCLH), University Medical Centre Hamburg‐Eppendorf, Maternal‐Foetal Unit Hospital Clinic Barcelona, and the Skåne University Hospital, Lund [10]. Before the study launched, obstetricians, foetal medicine specialists and neonatologists from the four academic health science centres compared the management of pregnancies affected by early onset FGR and the neonatal care. Differences in care, such as frequency of ultrasound scans, ultrasound indications for delivery and neonatal care were resolved so that there was consistent management across all sites [10]. Women aged 18 years and above with singleton pregnancies where estimated foetal weight was < 3rd centile for gestational age and < 600 g at 20 + 0 to 26 + 6 weeks of gestation were recruited to the study. Participants were excluded if, at enrolment, they had a known abnormal karyotype, major foetal structural abnormality, maternal HIV or hepatitis B or C infection, preterm prelabour rupture of membranes (PPROM), or indication for immediate delivery. The exclusion criteria extended to any medical or psychiatric condition which compromised the woman's ability to participate. Patients were involved in the design and analysis of the trial via the EVERREST Parents Advisory Group.

#### 2.1.2 Resource Use and Costs

An extensive data set was collected for each participant, which included the majority of the optimal data set collected by the Global Pregnancy CoLaboratory (COLAB) [11]. In terms of maternal resource use, this included records of ultrasound appointments, antenatal and postnatal maternal hospital stays. Information on the type of delivery was collected, and women were offered a postnatal review 6 weeks after birth to assess maternal outcomes. In terms of infant resource use, Neonatal Unit (NNU) stays were recorded during the initial hospitalisation after a live birth. A clinical researcher used detailed information on interventions and feeding received by infants to identify which days in the Neonatal Unit were intensive treatment (Neonatal Intensive Care Unit, NICU), high dependency (HDU), special care (SCBU) and transitional care (TC) [12]. Infant hospital, community service and infant medication data was collected up to 24 months after birth. Resource use was mapped onto NHS Reference Costs from 2019/20 [13] and Personal Social Service Research Unit (PSSRU) 2020 costs [14]. Medication was costed using the British National Formulary for Children (BNFc) [15].

For those cared for at UCLH, all participants were asked to complete an adapted Client Service Receipt Inventory (CSRI) [16] at 6 weeks post‐delivery, capturing costs and resource use from the time of diagnosis to completing the questionnaire. Women who had a liveborn baby were also asked to complete a second adapted CSRI at 6 months post‐delivery. Questions were asked regarding community service use, social service use, other hospital service use, reduced hours of work, unpaid carer hours and personal expenses. Data was costed using the top‐down approach and NHS Reference costs from 2019/20 [13] and PSSRU 2020 costs [14]. The human capital approach to productivity costs is used whereby the lost production time is multiplied by the relevant wage rate [17]. The product of the wage rate and the length of time absent from work was multiplied by 0.8 to reflect the likelihood that reduced work time results in a less than proportionate reduction in productivity [18]. A shadow price of a paid home care worker was used to reflect informal care, reflecting the assumption that if the informal carer could provide unpaid help, they could do the same in a paid professional capacity.

All costs are expressed in pounds sterling reflecting values for the financial year 2019/20, with all costs beyond the first year after birth discounted using the National Institute of Health and Care Excellence (NICE) recommended UK discount rate of 3.5% [19].

### 2.2 Stratification

The analysis is stratified by gestational age at delivery, according to the World Health Organization (WHO) categories: extremely preterm (EPT) (less than 28 + 0 weeks); very preterm (VPT) (28 + 0 to < 32 + 0 weeks); moderate to late preterm (MLPT) (32 + 0 to < 37 + 0 weeks); and term (37 + 0 weeks and above) [20]. Current management involves preterm delivery of the foetus before intrauterine death or irreversible organ damage occurs [21]. If stratification and significance tests indicate that preterm births are more costly than later term births, it provides economic justification for research investment into therapies that provide alternatives to standard of care. Furthermore, it is important to see whether it is the preterm nature of births or the FGR that is causing high costs. This cohort has no control group of preterm infants without FGR and, consequently, comparison with other studies that analyse more general preterm birth populations is the only way to isolate the impact of the severe early‐onset FGR. Stratification facilitates this comparison with other studies.

### 2.3 Statistical Analysis

Stata MP 15 was used to conduct statistical analyses. Basic descriptive statistics are reported to analyse differences in demographics between gestational age groups, with differences across groups tested for significance using ANOVA tests for continuous variables and chi‐squared tests for categorical variables.

The main results section focuses on resource use and costs derived from medical records only, due to 33.3% of UCLH mothers not filling in the 6‐week CSRI, and hence the main results are from a maternity and neonatal services cost perspective. This can partly be attributed to the introduction of the CSRI occurring after the EVERREST observational study start date, due to a delay in ethics approval for the CSRI. Information from patients whose 6‐week follow‐up fell before the CSRI's introduction could therefore not be captured. 6‐week CSRI costs are included as a secondary wider societal analysis, accounting for assumptions about missingness.

Statistics regarding population resource use are reported, namely the number and proportion of each group that used at least one unit of a service as well as the mean number of units used for that service for users. The mean values refer to length of stay (LOS) for inpatient services and contacts for outpatient services or appointments. The maternal population used to calculate percentages and means includes all eligible mothers, regardless of birth outcome. When considering infant resource use, only livebirths are included in the sample. The zero NNU and follow‐up costs associated with non‐livebirths would reduce the mean and misrepresent the costs associated with living with FGR complications. Moreover, other comparable studies only use livebirths in their infant samples [22, 23, 24].

Costs were split into antenatal maternal, postnatal maternal, delivery, NNU and infant follow‐up (includes infant medication) categories, to be used as dependent variables in models designed to test for the effect of gestational age at birth on costs. Significance tests were not performed for individual cost components to avoid the problems associated with multiple testing. All dependent variables had positively skewed distributions. To reduce the heteroscedasticity of regression residuals and hence violate the assumptions of linear regression, our regression models took the form of Generalised Linear Models (GLMs) with appropriate link functions [25]. After analysing log‐normal plots, Akaike Information Criterion (AIC) [26] and performing Park tests for heteroscedasticity, a gamma distribution and a log function were selected. Two models were used, one unadjusted and one adjusting for maternal and infant characteristics: site, maternal age, maternal ethnicity, parity, preeclampsia status, mode of conception, mode of delivery and birth outcome. Controlling for these variables also allowed for the identification of other cost drivers. The sex of the newborn was considered as a variable but removed because of missing data. When included, it had no impact on results. Only live births were considered for models analysing infant costs, with those who did not attend NNU incurring zero NNU costs. Death in NNU was adjusted for when calculating adjusted NNU cost differences for all live births. A further regression on NNU costs excluded all those who died in NNU to facilitate comparisons with other papers that looked at NNU survivors [22, 23, 24].

## 3 Results

### 3.1 Patient Characteristics

Table 1 details patient characteristics stratified by gestational age at birth. A cohort of 135 was available for analysis, with 64 born EPT, 31 VPT, 21 MLPT and 19 term. In terms of maternal characteristics, no significant differences were found between groups. For mode of delivery, differences were found, with significantly more Caesarean sections occurring in the later preterm groups, although this trend partially reversed in term group. This is likely due to the finding that earlier preterm groups suffered significantly more IUFDs and terminations of pregnancy which were managed by induction of labour; only 47% of EPT births resulted in a livebirth, compared with 100% of term births. The mean birthweight for the overall population was 1.14 kg (0.69 kg) and falls into the clinical category ‘very low birth weight’ [27], well below the UK average of 3.40 kg [28]. Even term infants weighed 2.23 kg (0.40 kg) on average, indicating that severe early‐onset FGR has an impact on birth weight independent of gestational age.

**TABLE 1:** Individual characteristics stratified by gestational age at birth.

	EPT (*n* = 64)	VPT (*n* = 31)	MLPT (*n* = 21)	Term (*n* = 19)
Maternal				
	***n* (%)**	***n* (%)**	***n* (%)**	***n* (%)**
White	37 (58)	15 (48)	14 (67)	11 (61)
IVF	12 (19)	5 (17)	1 (5)	0 (0)
Pregnancy induced hypertension	15 (25)	2 (7)	3 (17)	1 (6)
Pre‐eclampsia	13 (21)	7 (26)	4 (22)	1 (6)
HELLP syndrome	1 (2)	1 (4)	0 (0)	0 (0)
Gestational diabetes	1 (2)	1 (4)	2 (11)	0 (0)
Antenatal venous thromboembolism	0 (0)	2 (7)	0 (0)	0 (0)
Placenta praevia/low lying placenta	1 (2)	0 (0)	0 (0)	0 (0)
Antepartum haemorrhage	3 (5)	5 (19)	1 (6)	1 (6)
Placental abruption	1 (2)	1 (6)	1 (10)	1 (11)
	**Mean (SD)**	**Mean (SD)**	**Mean (SD)**	**Mean (SD)**
Maternal age (years)	34 (6.9)	33.3 (5.1)	33.7 (7.3)	33.6 (5.1)
Parity	1.3 (1.6)	1.3 (2.4)	1.8 (2.3)	1.7 (1.8)
Delivery				
	***n* (%)**	***n* (%)**	***n* (%)**	***n* (%)**
Mode of delivery				
Vaginal	36 (56)	7 (23)	1 (5)	7 (37)
Caesarean	28 (44)	24 (77)	19 (95)	12 (63)
Infant				
	***n* (%)**	***n* (%)**	***n* (%)**	***n* (%)**
Place of birth				
UCLH	45 (70)	27 (87)	20 (95)	16 (84)
Hamburg‐Eppen	6 (9)	3 (10)	0 (0)	0 (0)
Barcelona	8 (13)	1 (3)	1 (5)	2 (11)
Lund	5 (8)	0 (0)	0 (0)	1 (5)
Birth outcome				
Livebirth	30 (47)	26 (84)	20 (95)	19 (100)
IUFD	28 (44)	5 (16)	0 (0)	0 (0)
Termination of pregnancy	6 (9)	0 (0)	1 (5)	0 (0)
Infant death	9 (14)	3 (10)	0 (0)	1 (5)
	**Mean (SD)**	**Mean (SD)**	**Mean (SD)**	**Mean (SD)**
Birthweight (livebirths) (g)	507.1 (121.3)	818.4 (228.5)	1441.3 (342.1)	2235.2 (407.0)

*Note:* No patients experienced eclampsia, acute fatty liver or recurrent antepartum haemorrhage.

Abbreviations: EPT, extremely preterm infants; HELLP syndrome, syndrome of haemolysis, elevated liver enzymes and low platelets; IUFD, intrauterine fetal death/stillbirth; MLPT, moderate to late preterm infants; SD, standard deviation; VPT, very preterm infants.

### 3.2 Resource Use

Resource use from a maternity and neonatal services perspective for mothers and infants is presented in Table 2 and Tables S1–S3. 29 (97%), 25 (96%), 20 (100%) and 11 (58%) of infants spent at least one night in NNU in EPT, VPT, MLPT and term gestational age groups, for mean (standard deviation) lengths of stay of 112.4 (78.8), 83.9 (55.8), 27.9 (12.0) and 19.5 (29.6) days, respectively. Of these days, 28 (93%) and 22 (88%) of EPT and VPT infants spent at least one day in NNU, at an average of 46.4 (65.1) and 30.6 (58.6) days, respectively.

**TABLE 2:** Resource use from medical records, stratified by gestational age at birth.

	EPT (*n* = 64)		VPT (*n* = 31)		MLPT (*n* = 21)		Term (*n* = 19)	
	*n* (%)	Mean LOS or contacts (SD)	*n* (%)	Mean LOS or contacts (SD)	*n* (%)	Mean LOS or contacts (SD)	*n* (%)	Mean LOS or contacts (SD)
Maternal								
Antenatal inpatient	45 (70)	5.8 (5.6)	20 (65)	6.9 (6.7)	17 (81)	4.3 (4.1)	10 (53)	3.0 (1.1)
Antenatal outpatient	37 (58)	4.8 (2.7)	16 (52)	8.7 (6.4)	14 (67)	9.5 (5.7)	8 (42)	7.6 (6.1)
Antenatal ultrasounds	64 (100)	4.3 (2.0)	31 (100)	7.1 (3.0)	21 (100)	8.9 (2.6)	19 (100)	10.5 (2.7)
Postnatal hospital stays	37 (74)	3.1 (3.0)	19 (83)	3.5 (2.9)	12 (75)	3.8 (2.7)	14 (82)	7.7 (16.3)
	**EPT (*n* = 30)**		**VPT (*n* = 26)**		**MLPT (*n* = 20)**		**Term (*n* = 19)**	
Neonatal Unit (all infants)^a^	29 (97)	112.4 (78.8)	25 (96)	83.9 (55.8)	20 (100)	27.9 (12.0)	11 (58)	19.5 (29.6)
Neonatal Unit (survivors to discharge)^a^	22 (73)	135.3 (64.3)	23 (88)	89.3 (54.9)	20 (100)	27.9 (12.0)	11 (58)	19.5 (29.6)
Mean days in NICU^a^	28 (93)	46.4 (65.1)	22 (88)	30.6 (58.6)	9 (45)	3.9 (2.2)	4 (21)	26.8 (47.5)
Mean days in HDU^a^	21 (70)	73.9 (32.8)	22 (88)	29.2 (26.4)	16 (80)	7.9 (5.1)	3 (16)	4.7 (3.8)
Mean days in SCBU^a^	14 (47)	29.4 (27.7)	22 (88)	35.6 (13.7)	19 (95)	20.4 (10.0)	8 (42)	11 (12.2)
Mean days in TC^a^	0 (0)	0.0 (0.0)	0 (0)	0.0 (0.0)	1 (5)	7.0 (0.0)	2 (11)	3.0 (0.0)
Hospital appointments^b^	15 (68)	2.7 (2.4)	8 (35)	2.8 (1.7)	2 (10)	3.5 (0.7)	6 (55)	3.7 (3.4)
Non‐hospital appointments^b^	15 (68)	17.5 (9.2)	15 (65)	8.1 (4.3)	13 (65)	9.4 (6.5)	13 (118)	7.2 (3.8)

*Note:* Maternal resource use includes those with stillbirths and livebirths; Infant resource use is only presented for livebirths.

Abbreviations: EPT, extremely preterm infants; HDU, high dependency unit; LOS, length of stay; MLPT, moderate to late preterm infants; NICU, neonatal intensive care unit; SCBU, special care baby unit; SD, standard deviation; TC, transitional care; VPT, very preterm infants.

^a^ Only those who stayed in the neonatal unit were included in the sample.

^b^ Only those who stayed in the neonatal unit and survived to discharge were included in the sample.

### 3.3 Costs

Tables 3 and 4 report mean maternity services costs by gestational age at birth, unadjusted and adjusted, respectively. NNU accounts for the largest costs incurred by either mother or infant and shows the most pronounced differences between gestational age groups, with all preterm groups having significantly larger costs when adjusting for maternal and infant variables. Compared with term births, preterm births have significantly higher NNU costs, with EPT infants costing an additional £157 771 (95% CI: £96 974–£218 668) on average per infant compared with the term group, VPT infants an additional £93 343 (95% CI: £62 387–£124 300) and MLPT infants an additional £20 195 (£11 886–£28 503). The EPT group was also significantly more expensive in terms of infant follow‐up costs.

**TABLE 3:** Medical records unadjusted mean costs and unadjusted difference in means from GLMs using term group as the comparator, stratified by gestational age at birth.

		Mean (SE) (£)	Mean difference (£) (95% CI)	*p*
Antenatal maternal (*n* = 135)	Term	11 625 (1496)		
	MLPT	17 138 (3090)	5513 (−1216 to 12 242)	0.108
	VPT	18 250 (3287)	6625 (−453 to 13 703)	0.067
	EPT	14 563 (2054)	2938 (−2043 to 7918)	0.248
Postnatal maternal (*n* = 106)	Term	15 566 (9177)		
	MLPT	6733 (2319)	−8833 (−27 384 to 9718)	0.351
	VPT	7612 (2119)	−7955 (−26 414 to 10 505)	0.398
	EPT	5768 (1369)	−9798 (−27 983 to 8387)	0.291
Delivery (*n* = 128)	Term	5042 (320)		
	MLPT	5327 (319)	285 (−601 to 1172)	0.528
	VPT	5183 (276)	141 (−687 to 970)	0.739
	EPT	4639 (183)	−403 (−1127 to 320)	0.275
NNU (*n* = 95)	Term	14 029 (8643)		
	MLPT	24 409 (2457)	10 380 (−7231 to 27 991)	0.248
	VPT	92 748 (17 730)	78 719 (40 060 to 117 378)	0.000
	EPT	140 755 (21 671)	126 726 (80 998 to 172 453)	0.000
NNU (survivors to discharge) (*n* = 86)	Term	14 029 (8648)		
	MLPT	24 409 (2458)	10 380 (−7241 to 28 001)	0.248
	VPT	97 222 (18 921)	83 193 (42 419 to 123 967)	0.000
	EPT	165 101 (23 150)	151 071 (102 637 to 199 506)	0.000
Infant follow‐up (*n* = 86)	Term	3074 (866)		
	MLPT	2330 (560)	−744 (−2766 to 1278)	0.471
	VPT	2757 (637)	−317 (−2424 to 1789)	0.768
	EPT	5726 (1136)	2651 (−148 to 5450)	0.063

*Note:* Only livebirths are included in the NNU, those infants that were not admitted to the NNU are included as zero cost. Only livebirths that did not die in NNU were included in infant‐follow‐up rgeressions. Livebirths that did not die in NICU and did not report any follow‐up admissions or appointments were included as zero cost. Infant follow‐up includes hospital readmission and health professional appointment costs.

Abbreviations: EPT, extremely preterm infants; GLM, generalised linear model; LOS, length of stay; MLPT, moderate to late preterm infants; NNU, neonatal unit; SE, standard error; VPT, very preterm infants.

**TABLE 4:** Medical records adjusted mean costs and adjusted mean differences from GLMs using term group as the comparator, stratified by gestational age at birth, adjusting for recruitment site, maternal age, maternal ethnicity, parity, preeclampsia status, mode of conception, mode of delivery, birth outcome^a^ and death in NNU^b^.

		Mean (SE) (£)	Mean difference (£) (95% CI)	*p*
Antenatal maternal (*n* = 117)	Term	12 658 (1785)		
	MLPT	21 359 (3815)	8700 (775 to 16 626)	0.031
	VPT	21 020 (3465)	8361 (249 to 16 474)	0.043
	EPT	14 941 (1791)	2283 (−3428 to 7994)	0.433
Postnatal maternal (*n* = 100)	Term	6811 (2679)		
	MLPT	6311 (3081)	−500 (−7416 to 6416)	0.887
	VPT	8701 (3293)	1890 (−4487 to 8267)	0.561
	EPT	13 257 (5408)	6447 (−3504 to 16 398)	0.204
Delivery (*n* = 111)	Term	5601 (174)		
	MLPT	4946 (209)	−655 (−1151 to −158)	0.010
	VPT	5115 (114)	−486 (−870 to −102)	0.013
	EPT	5131 (117)	−469 (−940 to 2)	0.051
NNU (*n* = 81)	Term	4872 (2457)		
	MLPT	25 067 (3887)	20 195 (11 886 to 28 503)	0.000
	VPT	98 216 (15 940)	93 343 (62 387 to 124 300)	0.000
	EPT	162 643 (30 964)	157 771 (96 874 to 218 668)	0.000
NNU (survivors to discharge) (*n* = 72)	Term	5409 (2770)		
	MLPT	26 205 (3548)	20 796 (12 809 to 28 782)	0.000
	VPT	105 908 (20 097)	100 499 (61 228 to 139 769)	0.000
	EPT	170 433 (32 243)	165 024 (101 455 to 228 594)	0.000
Infant follow‐up (*n* = 72)	Term	4529 (1464)		
	MLPT	2970 (715)	−1559 (−4719 to 1601)	0.334
	VPT	3212 (795)	−1318 (−4707 to 2072)	0.446
	EPT	4728 (905)	199 (−3204 to 3602)	0.909

*Note:* Only livebirths are included in the NNU, those infants that were not admitted to the NNU are included as zero cost. Only livebirths that did not die in NNU were included in infant‐follow‐up regressions. Livebirths that did not die in NICU and did not report any follow‐up admissions or appointments were included as zero cost. Infant follow‐up includes hospital readmission and health professional appointment costs.

Abbreviations: EPT, extremely preterm infants; GLM, generalised linear model; LOS, length of stay; MLPT, moderate to late preterm infants; NNU, neonatal unit; SE, standard error; VPT, very preterm infants.

^a^ Only included for antenatal maternal, postnatal maternal and delivery costs since infant cost regressions were restricted to livebirths.

^b^ Only included in NNU regression.

In the unadjusted model when compared with the term group, the antenatal maternal costs are not significantly higher in any preterm group. When adjustment variables are included, these maternal costs are significantly higher in both the MLPT preterm group and VPT group, at adjusted differences of £8700 (95% CI: £775–£16 626) and £8361 (95% CI: £249–£16 474), respectively. Postnatal maternal stays are not significantly different for any groups in either model.

When adjustment variables are included in models, the n reduces by between 5.67% and 16.28%, potentially biasing estimates. The adjusted regression models' output is included in the appendix (Tables S6 and S7).

### 3.4 Secondary Analysis

No observable predictors of missingness were found for the 6‐week CSRI and we assume that the missingness is not systematically related to the unobserved data. Women experiencing a stillbirth were not routinely offered a 6‐month postnatal appointment and therefore only one mother who did not have a live birth completed the 6‐month CSRI.

No significant differences were found in the unadjusted regressions based on the 6‐week CSRI, where costs are reported in Table S4. After adjustment, reported in Table S5, the EPT group had significantly lower community costs than the term group, at an adjusted difference of −£338 (95% CI: −£660 to −£15). Costs beyond health and social care were the most expensive CSRI costs across all gestational ages.

### 3.5 Other Predictors

The adjusted GLM output is displayed in Tables S6 and S7. Preeclampsia was associated with significantly higher antenatal inpatient and postnatal inpatient maternal costs. White mothers had significantly lower antenatal maternal costs but their infants had significantly higher follow‐up costs. For NNU costs, death in NNU was a significant predictor of a lower cost, with an adjusted difference of −£92 055 compared to survivors. Those treated at Hamburg‐Eppendorf had significantly higher antenatal maternal costs at an adjusted difference of £21 027, but significantly lower NNU costs at an adjusted difference of −£54 118, when compared with UCLH. This may be due to the different clinical practices of inpatient monitoring (at Hamburg‐Eppendorf) compared with out‐patient monitoring (at UCLH) for women with early onset FGR.

## 4 Discussion

### 4.1 Main Findings

In this prospective study of pregnancies complicated by early‐onset FGR before 27 weeks of gestation we found substantial economic impact. Costs were predominantly incurred during infants' NNU admissions, with births < 32 + 0 weeks having significantly higher costs than term births. This paper fills an important gap in a sparse body of literature concerning the economic cost of severe early‐onset FGR, an area in which drug development is increasingly being focussed [29]. Our main findings concern two key points. First, we find that maternal resource use and costs are higher in some categories for preterm groups when compared with the term group. The majority of costs, however, come from long and intensive infant inpatient stays in NNU, particularly NICU, following iatrogenic preterm birth. All preterm birth groups have significantly larger NNU costs, a consequential finding because current management of FGR involves early delivery, which evidently comes at a substantial cost, partly due to the complications associated with prematurity. A recent analysis comparing NNU length of stay and neonatal management found that compared with appropriately grown gestational‐age matched neonates, those with severe early‐onset FGR had a higher risk of respiratory morbidity, surgical necrotizing enterocolitis and treatment for retinopathy of prematurity, and experienced a prolonged duration of ventilation, delayed establishing of enteral feeds, poor postnatal growth and delayed discharge [12]. The development of novel interventions that can delay the necessity of early delivery in FGR may generate substantial cost savings. The economic benefit of preventing preterm birth extends to those who are born MLPT. Future research could explore if there is an ideal gestational age of delivery for FGR that balances infant short‐term and long‐term outcomes, cost and the health of the mother.

Second, it is beneficial to know whether this exclusively severe early‐onset FGR population has economic costs larger than more general populations of preterm born infants. Comparisons with other preterm studies enable us to assess the impact of FGR. Khan et al. [22] found term births and MLPT births had average costs to discharge of £2296 and £6815, after applying the HCHS and NHSCII price inflation indices to obtain 2019/20 prices. This compares to costs that were £4872 for term births and £25 067 for MLPT infants in our analysis, suggesting that even for MLPT infants, FGR is associated with additional costs. When analysing a cohort of infants born preterm in England and Wales, Mangham et al. [23] found neonatal costs of £73 109 and £106 958 for survivors who had a VPT birth or EPT birth, respectively, after adjusting for inflation. This also appears lower than our comparable estimates of £98 216 and £162 643, respectively, indicating that FGR gives rise to costs over and above those caused by preterm birth.

Using length of stay as the comparative outcome overcomes concerns regarding the comparison of studies that use alternative model specifications and costing methods. Seaton et al. [24] provide an international comparison of length of stay for infants born EPT. When analysing survivors only, they report a mean length of stay of 82 days for the UK and 87 days for all included countries. Data from another study by Seaton et al. [30] found mean length of stay for the VPT group and EPT group was 46 and 96 days. Again, these are lower than the values of 89 and 135 days calculated in our analysis, which could be due to the focus on EPT with early‐onset FGR in this EVERREST cohort when compared to those studies that included all EPT births. These are simplistic comparisons but warrant further systematic exploration.

### 4.2 Strengths and Limitations

The main strength of this study lies in the fact that it is the first of its kind to assess the economic costs of a population with severe early‐onset FGR. This population has not been explicitly identified in previous studies, so it has not been possible to analyse the costs that are attributable to severe early‐onset FGR specifically. The methods used and display of results facilitate comparisons with other studies and thus enable cautious conclusions to be drawn regarding the economic severity of FGR. The methodology uses rigorous costing methods that follow national guidance for health economic evaluation purposes. The findings are also invaluable for drug development purposes. FGR due to placental insufficiency has achieved orphan drug status, which provides some cost savings for drug developers [31]. To take a novel drug to market, however, also requires knowledge of the healthcare burden and costs of the condition to be targeted. These findings provide this information, allowing the potential market value and pricing of a novel intervention to improve outcome in severe early‐onset FGR to be better understood.

The study consists of several limitations. The sample size, particularly for subgroup analysis, was small and with heavily positively skewed costs, which led to large standard errors and wide confidence intervals when calculating statistics. Furthermore, the study drew participants predominantly from UCLH in London, with smaller numbers participating from elsewhere in Europe and may not be representative of the wider UK or European population, where regional differences in care pathways and patient demographics may impact costs. Further larger studies of more diverse populations are needed to address generalisability. This study also only considers costs up to 2 years after birth. Other studies have considered the long‐term cost implications of preterm births. Petrou et al. [32] found that public sector cost for 11‐year‐olds who were born EPT were £6484 during the 11th year of life, compared with £4007 for a term control group, at a mean difference of £2477. Future research should aim to establish whether these differences persist for a population with FGR and whether they are above that of a broader preterm group.

In addition, using regression analysis to isolate the influence of FGR is not possible since there is no non‐FGR comparison group. To overcome this, we have compared our results with other similar studies in the literature that have used all preterm births, noting that this may include some FGR cases. Any conclusions drawn from this should be approached with caution as the year of study, costing methods, regression variables, sample characteristics and other inputs may differ substantially. As no information was available on the socioeconomic status of the mothers, we were unable to adjust for any potential effects this may have had on outcomes.

Future research should focus on collecting more complete data for the broader health service and societal costs post discharge. The CSRI had poor follow‐up rates, particularly amongst mothers who did not have a livebirth, and relies on patient recall, a technique known to be unreliable in certain situations. Only livebirths were included in the evaluation of neonatal costs in line with the other literature in this area. This means that the wider societal cost of a death is not incorporated into the analysis, potentially underestimating the total cost.

### 4.3 Interpretation

Cost of illness studies are criticised as they do not offer insights into how the reported cost may be reduced through cost effective interventions (Supporting Information) [33]. However, this study has shown that the proposed EVERREST Ad.VEGF therapy has potential to substantially reduce costs if it reduces the number of preterm births and mitigates the extra costs of FGR through delaying preterm birth and increasing foetal growth.

## 5 Conclusion

Mothers and infants with severe early onset FGR incur substantial costs from the start of pregnancy through to two years after birth. Most of these costs are attributed to the infants' NNU stay. Younger gestational ages are associated with larger costs, a finding regularly seen in the literature on preterm births. Since preterm births are extremely common in this population, this finding provides economic justification for research into the development of therapies that may delay early delivery and improve foetal growth. Changes in policy regarding the clinical care and management decisions for women with early onset FGR should include an assessment of the economic impact as this may be substantial. Further research should systematically explore whether FGR leads to increased costs over and above those associated with preterm populations without FGR.

## Author Contributions

G.B., R.H. and A.L.D. contributed to data analysis and writing the manuscript. R.N.S., D.M.P., D.S., K.H., A.D., S.R.H., F.F., E.G., F.C. and A.L.D. contributed to study design, patient recruitment and data collection. M.D., K.M., Y.G. and T.W. contributed to patient recruitment and data collection. G.B. and J.O. contributed to data collection. N.M. and A.H.‐C. contributed to the study design. All authors contributed to critically revising the manuscript, giving approval for publication of the work and agreeing to be accountable for all aspects of it.

## Ethics Statement

Ethical approval was provided on 17th September 2013 by the National Research Ethics Service Committee London–Stanmore in the UK (REC reference: 13/LO/1254), the Hospital Clinic of Barcelona's Clinical Research Ethics Committee in Spain (Reg: HCB/2014/0091), the Regional Ethical Review Board in Lund for Sweden (DNr 2014/147) and the Ethics Committee of Hamburg Board of Physicians in Germany (PV4809). This study was conducted according to the Declaration of Helsinki principles, and written informed consent was given by all participants before enrolment.

## Conflicts of Interest

The authors declare no conflicts of interest.

## Supporting information


Appendix S1.

Table S1.–S7.

## Data Availability

The full data set will not be made publicly available because the degree of detailed phenotyping could allow individual patient identification. Limited data sharing may be possible, with the agreement of the consortium, on request to R.H. or A.L.D.
