# Supplementary material for: Economic Impact of Severe Early‐Onset Foetal Growth Restriction: A Multicentre Prospective Cohort Study
Source: BJOG. 2025 Jun 24;133(1):61–70. doi: 10.1111/1471-0528.18266 (PMC12676201; doi:10.1111/1471-0528.18266)
Supplement: Supplementary file 1 — Appendix S1. [file BJO-133-61-s001.docx]

cost of illness checklist (34).

| **Item** | **Question** | **Answer** | **Supportive information** |
| --- | --- | --- | --- |
| Study characteristics | |  |  |
| Question/objective | 1. Is a well-defined research question or objective stated? | Yes |  |
| Population | 1. Is the study population described? | Yes |  |
| Perspective | 1. a) Is (are) the chosen study perspective(s) stated? | Yes |  |
|  | b) If so, is (are) the chosen study perspective(s) justified? | Yes |  |
| Methodology and cost analysis | |  |  |
| Epidemiological approach | 1. Is the epidemiological approach reported (e.g., prevalence, incidence)? | N/A | Not appropriate given not a cost of illness study. |
| Costing approach | 1. Is the costing approach reported (e.g., top-down, bottom-up)? | Yes |  |
| Data collection approach | 1. Is the data collection process reported (e.g., prospective, retrospective)? | Yes |  |
| Identification | 1. a) Are all components of resource use identified that are relevant to the condition/disease, population, intervention, study objectives, and study perspective? | Yes |  |
|  | b) If not, is a justification provided for excluding relevant components of resource use? | N/A |  |
| Measurement | 1. a) Are all included components of resource use measured? | Yes |  |
|  | b) If not, is a justification provided for not measuring certain components of resource use? | N/A |  |
| Valuation | 1. a) Are all included components of resource use valued in monetary terms? | Yes |  |
|  | b) If not, is a justification provided for not valuing certain components of resource use? | N/A |  |
| Time horizon | 1. a) Is the chosen time horizon specified? | Yes |  |
|  | b) If so, is the chosen time horizon justified? | Yes |  |
| Discounting | 1. a) Are future costs discounted? | Yes |  |
|  | b) If so, is a justification provided for the discount rate? | Yes |  |
| Sensitivity | 1. a) Are all variables whose values are uncertain subjected to sensitivity analysis? | Yes |  |
|  | b) If so, is a justification provided for which variables are subjected to sensitivity analysis? | Yes |  |
|  | c) Are analyses done on relevant subgroups? | Yes |  |
| Results and reporting | |  |  |
| Cost sectors | 1. Are the study results presented transparently by cost category/sector? | Yes |  |
| Generalizability | 1. Do the authors discuss the generalizability of study results (e.g., comparing the results to other patient/client groups or/in other settings)? | Yes |  |
| Limitations | 1. Do the authors discuss important limitations? | Yes |  |
| Ethical and distributional issues | 1. a) Do the authors discuss ethical issues? | Yes |  |
|  | b) Do the authors discuss distributional issues? | Yes |  |
| Conflict of interest | 1. Do the authors report any potential conflicts of interest? | Yes | None to report |
