# Supplementary material for: Economic Impact of Severe Early‐Onset Foetal Growth Restriction: A Multicentre Prospective Cohort Study
Source: BJOG. 2025 Jun 24;133(1):61–70. doi: 10.1111/1471-0528.18266 (PMC12676201; doi:10.1111/1471-0528.18266)
Supplement: Supplementary file 2 — Table S1.–S7. [file BJO-133-61-s002.docx]

**Table S1.** Maternal resource use from 6-week CSRI, stratified by gestational age at birth.

|  | EPT (n=62) | | VPT (n=31) | | MLPT (n=21) | | Term (n=19) | |
| --- | --- | --- | --- | --- | --- | --- | --- | --- |
|  | n (%) | Mean LOS or contacts (SD) | n (%) | Mean LOS or contacts (SD) | n (%) | Mean LOS or contacts (SD) | n (%) | Mean LOS or contacts (SD) |
| Community services |  |  |  |  |  |  |  |  |
| GP surgery | 24 (73) | 2.8 (1.9) | 10 (63) | 3.3 (2.1) | 6 (60) | 2.3 (1) | 12 (92) | 2.2 (1.9) |
| GP phone | 5 (15) | 2.2 (0.8) | 2 (13) | 1 (0) | 2 (20) | 1.5 (0.7) | 3 (23) | 1 (0) |
| GP practice nurse | 4 (12) | 1.3 (0.5) | 4 (25) | 1.8 (1) | 2 (20) | 1 (0) | 2 (15) | 1 (0) |
| GP practice nurse phone | 1 (3) | 1 (0) | 0 (0) | 0 (0) | 1 (10) | 1 (0) | 0 (0) | 0 (0) |
| Community midwife | 21 (64) | 2.8 (2.4) | 8 (50) | 3.1 (1.8) | 3 (30) | 4.7 (4.6) | 7 (54) | 2.6 (1.3) |
| Community antenatal clinic | 4 (12) | 5 (4.8) | 1 (6) | 5 (0) | 0 (0) | 0 (0) | 2 (15) | 1.5 (0.7) |
| Health Visitor | 2 (6) | 1 (0) | 1 (6) | 1 (0) | 0 (0) | 0 (0) | 2 (15) | 5.5 (3.5) |
| Health visitor at home | 8 (24) | 2.3 (1.2) | 8 (50) | 3.1 (2.1) | 7 (70) | 3 (1.5) | 9 (69) | 2.8 (1.1) |
| Counsellor/ CBT therapist | 6 (19) | 2.6 (2.8) | 0 (0) | 0 (0) | 1 (10) | 1.5 (0) | 1 (8) | 2 (0) |
| Physiotherapist | 1 (3) | 2 (0) | 1 (6) | 5 (0) | 0 (0) | 0 (0) | 1 (8) | 1 (0) |
| Social services |  |  |  |  |  |  |  |  |
| Social worker | 0 (0) | 0 (0) | 0 (0) | 0 (0) | 1 (10) | 5 (0) | 0 (0) | 0 (0) |
| Social worker (phone call) | 0 (0) | 0 (0) | 0 (0) | 0 (0) | 1 (10) | 4 (0) | 0 (0) | 0 (0) |
| Hospital services |  |  |  |  |  |  |  |  |
| Hospital midwife telephone | 12 (36) | 3.3 (1.9) | 3 (19) | 3 (1) | 1 (10) | 3 (0) | 3 (23) | 3.7 (1.5) |
| Maternity assessment centre | 11 (33) | 2.4 (3.2) | 6 (38) | 1.7 (0.5) | 2 (20) | 5.8 (6) | 7 (54) | 5 (8.5) |
| Breast feeding support | 0 (0) | 0 (0) | 2 (13) | 1.5 (0.7) | 1 (10) | 2 (0) | 0 (0) | 0 (0) |
| Ambulance | 2 (6) | 2 (0) | 5 (31) | 5.4 (5.2) | 2 (20) | 1.5 (0.7) | 3 (23) | 2.3 (1.5) |
| A&E | 4 (12) | 1 (0) | 2 (13) | 1.5 (0.7) | 1 (10) | 1 (0) | 2 (15) | 1 (0) |
| Societal resource use |  |  |  |  |  |  |  |  |
| Reduced working | 11 (34) | 15.7 (13.7) | 7 (44) | 21.4 (15.6) | 4 (50) | 10.8 (3) | 3 (23) | 14.7 (12.2) |
| Unpaid care | 14 (61) | 17.9 (20.7) | 8 (53) | 22 (26.5) | 6 (75) | 37.3 (64.8) | 6 (55) | 21.3 (22.2) |

EPT = Extremely preterm infants, VPT = Very preterm infants, MLPT = Moderate to late preterm infants, LOS = Length of stay, A&E = Accident and emergency

**Table S2.** Maternal resource use from 6-month CSRI, stratified by gestational age at birth.

|  | EPT (n=62) | | VPT (n=31) | | MLPT (n=21) | | Term (n=19) | |
| --- | --- | --- | --- | --- | --- | --- | --- | --- |
|  | n (%) | Mean LOS or contacts (SD) | n (%) | Mean LOS or contacts (SD) | n (%) | Mean LOS or contacts (SD) | n (%) | Mean LOS or contacts (SD) |
| Community services |  |  |  |  |  |  |  |  |
| GP surgery | 1 (25) | 1 (0) | 2 (25) | 1.5 (0.7) | 3 (43) | 1.3 (0.6) | 0 (0) | 0 (0) |
| GP phone | 0 (0) | 0 (0) | 0 (0) | 0 (0) | 0 (0) | 0 (0) | 0 (0) | 0 (0) |
| GP practice nurse | 0 (0) | 0 (0) | 1 (13) | 1 (0) | 1 (14) | 2 (0) | 0 (0) | 0 (0) |
| GP practice nurse phone | 0 (0) | 0 (0) | 0 (0) | 0 (0) | 0 (0) | 0 (0) | 0 (0) | 0 (0) |
| Community midwife | 1 (25) | 2 (0) | 1 (13) | 2 (0) | 1 (14) | 3 (0) | 0 (0) | 0 (0) |
| Community antenatal clinic | 0 (0) | 0 (0) | 0 (0) | 0 (0) | 1 (14) | 10 (0) | 0 (0) | 0 (0) |
| Health Visitor | 0 (0) | 0 (0) | 0 (0) | 0 (0) | 0 (0) | 0 (0) | 0 (0) | 0 (0) |
| Health visitor at home | 0 (0) | 0 (0) | 0 (0) | 0 (0) | 0 (0) | 0 (0) | 0 (0) | 0 (0) |
| Counsellor/ CBT therapist | 0 (0) | 0 (0) | 1 (13) | 3 (0) | 2 (29) | 2 (1.4) | 0 (0) | 0 (0) |
| Physiotherapist | 0 (0) | 0 (0) | 0 (0) | 0 (0) | 0 (0) | 0 (0) | 0 (0) | 0 (0) |
| Social services |  |  |  |  |  |  |  |  |
| Social worker | 0 (0) | 0 (0) | 0 (0) | 0 (0) | 1 (14) | 2 (0) | 0 (0) | 0 (0) |
| Social worker (phone call) | 0 (0) | 0 (0) | 0 (0) | 0 (0) | 1 (14) | 2 (0) | 0 (0) | 0 (0) |
| Hospital services |  |  |  |  |  |  |  |  |
| General hospital ward | 0 (0) | 0 (0) | 0 (0) | 0 (0) | 0 (0) | 0 (0) | 0 (0) | 0 (0) |
| Delivery suite | 0 (0) | 0 (0) | 0 (0) | 0 (0) | 0 (0) | 0 (0) | 0 (0) | 0 (0) |
| Postnatal ultrasound | 1 (25) | 4 (0) | 1 (13) | 2 (0) | 1 (14) | 1 (0) | 0 (0) | 0 (0) |
| Hospital midwife telephone | 1 (25) | 6 (0) | 1 (13) | 6 (0) | 1 (14) | 10 (0) | 0 (0) | 0 (0) |
| Ambulance | 1 (25) | 2 (0) | 0 (0) | 0 (0) | 0 (0) | 0 (0) | 0 (0) | 0 (0) |
| Societal resource use |  |  |  |  |  |  |  |  |
| Reduced working | 0 (0) | 0 (0) | 0 (0) | 0 (0) | 0 (0) | 0 (0) | 0 (0) | 0 (0) |
| Unpaid care | 2 (50) | 6.5 (4.9) | 3 (43) | 17 (20.1) | 2 (33) | 21.5 (26.2) | 1 (33) | 2.5 (0) |
| Maternity leave | 1 (25) | - | 4 (50) | - | 5 (71) | - | 1 (33) | - |

EPT = Extremely preterm infants, VPT = Very preterm infants, MLPT = Moderate to late preterm infants, LOS = Length of stay, GP = General Practitioner, CBT = Cognitive behavioural therpaist

**Table S3.** Infant resource use from 6-month CSRI, stratified by gestational age at birth.

|  | EPT (n=62) | | VPT (n=31) | | MLPT (n=21) | | Term (n=19) | |
| --- | --- | --- | --- | --- | --- | --- | --- | --- |
|  | n (%) | Mean LOS or contacts (SD) | n (%) | Mean LOS or contacts (SD) | n (%) | Mean LOS or contacts (SD) | n (%) | Mean LOS or contacts (SD) |
| Community services |  |  |  |  |  |  |  |  |
| GP surgery | 1 (25) | 1 (0) | 4 (50) | 1.5 (1) | 4 (57) | 1.5 (0.6) | 2 (67) | 4.5 (2.1) |
| GP phone | 0 (0) | 0 (0) | 1 (13) | 1 (0) | 1 (14) | 1 (0) | 1 (33) | 2 (0) |
| GP practice nurse | 0 (0) | 0 (0) | 0 (0) | 0 (0) | 1 (14) | 1 (0) | 1 (33) | 1 (0) |
| GP practice nurse phone | 0 (0) | 0 (0) | 0 (0) | 0 (0) | 0 (0) | 0 (0) | 0 (0) | 0 (0) |
| Community midwife | 0 (0) | 0 (0) | 1 (13) | 1 (0) | 0 (0) | 0 (0) | 0 (0) | 0 (0) |
| Health Visitor | 1 (25) | 1 (0) | 3 (38) | 3.3 (0.6) | 3 (43) | 3 (2.6) | 1 (33) | 5 (0) |
| Health visitor at home | 0 (0) | 0 (0) | 1 (13) | 1 (0) | 2 (29) | 1.5 (0.7) | 0 (0) | 0 (0) |
| Hospital services |  |  |  |  |  |  |  |  |
| General hospital ward | 2 (50) | 1 (0) | 0 (0) | 0 (0) | 1 (14) | 1 (0) | 0 (0) | 0 (0) |
| Intensive care | 0 (0) | 0 (0) | 0 (0) | 0 (0) | 1 (14) | 1 (0) | 0 (0) | 0 (0) |
| Neonatal nurse phone | 0 (0) | 0 (0) | 0 (0) | 0 (0) | 0 (0) | 0 (0) | 0 (0) | 0 (0) |
| Ambulance | 0 (0) | 0 (0) | 0 (0) | 0 (0) | 1 (14) | 1 (0) | 0 (0) | 0 (0) |
| A&E | 1 (25) | 1 (0) | 1 (13) | 1 (0) | 2 (29) | 1 (0) | 0 (0) | 0 (0) |

EPT = Extremely preterm infants, VPT = Very preterm infants, MLPT = Moderate to late preterm infants, LOS = Length of stay, GP = General practitioner, A&E = Accident and emergency

**Table S4.** 6-week CSRI unadjusted mean costs and unadjusted mean differences from GLMs using term group as the comparator, stratified by gestational age at birth.

|  |  | Mean (SE) (£) | Mean difference (£) | p-value |
| --- | --- | --- | --- | --- |
| Community services (n = 72) | Term | 529 (106) |  |  |
|  | MLPT | 424 (110) | -105 (-405 to 195) | 0.494 |
|  | VPT | 430 (89) | -99 (-371 to 173) | 0.476 |
|  | EPT | 355 (60) | -174 (-414 to 66) | 0.155 |
| Hospital services (n = 72) | Term | 931 (600) |  |  |
|  | MLPT | 452 (329) | -479 (-1,820 to 862) | 0.484 |
|  | VPT | 371 (75) | -561 (-1,745 to 623) | 0.353 |
|  | EPT | 321 (129) | -611 (-1,813 to 591) | 0.319 |
| Societal costs (n = 68) | Term | 2,973 (1,265) |  |  |
|  | MLPT | 6,738 (4,099) | 3,765 (-4,642 to 12,172) | 0.38 |
|  | VPT | 4,474 (1,238) | 1,500 (-1,968 to 4,968) | 0.397 |
|  | EPT | 3,122 (878) | 148 (-2,869 to 3,165) | 0.923 |

GLM = Generalized Linear Model, SE = Standard error, EPT = Extremely preterm infants, VPT = Very preterm infants, MLPT = Moderate to late preterm infants, LOS = Length of stay

**Table S5.** 6-week CSRI adjusted mean costs and adjusted mean differences from GLMs using term group as the comparator, stratified by gestational age at birth, adjusting for site, maternal age, maternal ethnicity, parity, preeclampsia status, mode of conception, mode of delivery, birth outcome* and death in NNU**.

|  |  | Mean (SE) (£) | Mean difference (£) | p-value |
| --- | --- | --- | --- | --- |
| Community services (n = 63) | Term | 708 (182) |  |  |
|  | MLPT | 529 (193) | -122 (-429 to 185) | 0.435 |
|  | VPT | 419 (129) | -219 (-511 to 72) | 0.141 |
|  | EPT | 316 (65) | -338 (-660 to -15) | 0.040 |
| Hospital services (n = 63) | Term | 2,520 (2,229) |  |  |
|  | MLPT | 354 (391) | -1,052 (-3,228 to 1,123) | 0.343 |
|  | VPT | 428 (171) | -951 (-2,371 to 470) | 0.190 |
|  | EPT | 302 (133) | -1,137 (-2,719 to 445) | 0.159 |
| Societal costs (n = 59) | Term | 5,384 (4,581) |  |  |
|  | MLPT | 10,604 (12,138) | 3,580 (-6,253 to 13,414) | 0.475 |
|  | VPT | 3,366 (1,033) | -2,480 (-13,212 to 8,253) | 0.651 |
|  | EPT | 4,984 (2,725) | -407 (-11,580 to 10,766) | 0.943 |

GLM = Generalized Linear Model, SE = Standard error, EPT = Extremely preterm infants, VPT = Very preterm infants, MLPT = Moderate to late preterm infants, LOS = Length of stay

*Only included for antenatal maternal, postnatal maternal and delivery costs since infant cost regressions were restricted to livebirths.

**Only included in NNU regression.

**Table S6.** GLM adjusted regression output for costs calculated using medical record resource use.

|  | Antenatal maternal | Postnatal maternal | Delivery | NNU | NNU (survivors only) | Infant follow-up |
| --- | --- | --- | --- | --- | --- | --- |
| Extremely preterm | 2283.2 | 6446.6 | -469.3 | 157770.7*** | 165024.2^***^ | 199.1 |
|  | (0.433) | (0.204) | (0.051) | (0.000) | (0.000) | (0.909) |
|  |  |  |  |  |  |  |
| Very preterm | 8361.4* | 1889.9 | -486.0* | 93343.5*** | 100498.8^***^ | -1317.6 |
|  | (0.043) | (0.561) | (0.013) | (0.000) | (0.000) | (0.446) |
|  |  |  |  |  |  |  |
| Late preterm | 8700.5* | -500.1 | -654.6** | 20194.7*** | 20795.6^***^ | -1559.1 |
|  | (0.031) | (0.887) | (0.010) | (0.000) | (0.000) | (0.334) |
|  |  |  |  |  |  |  |
| Term | 0 | 0 | 0 | 0 | 0 | 0 |
|  | (.) | (.) | (.) | (.) | (.) | (.) |
|  |  |  |  |  |  |  |
| Maternal age at enrolment | 209.6 | 330.9 | 14.38 | -67.63 | -221.6 | 148.5 |
|  | (0.378) | (0.225) | (0.282) | (0.963) | (0.897) | (0.254) |
|  |  |  |  |  |  |  |
| White | -7175.6* | -752.6 | 165.6 | -14288.5 | -11197.0 | 2110.5 |
|  | (0.017) | (0.804) | (0.297) | (0.579) | (0.688) | (0.058) |
|  |  |  |  |  |  |  |
| Parity | 133.0 | 2284.1 | 33.72 | -30.72 | -1613.6 | 7.276 |
|  | (0.830) | (0.182) | (0.140) | (0.994) | (0.684) | (0.964) |
|  |  |  |  |  |  |  |
| University College Hospital London | 0 | 0 | 0 | 0 | 0 | 0 |
|  | (.) | (.) | (.) | (.) | (.) | (.) |
|  |  |  |  |  |  |  |
| University Medical Center Hamburg-Eppendorf | 21027.1** | 9981.6 | -90.54 | -54117.6** | -53202.5^**^ | 5629.1 |
|  | (0.005) | (0.190) | (0.737) | (0.004) | (0.006) | (0.120) |
|  |  |  |  |  |  |  |
| Maternal-Fetal Unit Hospital Clinic de Barcelona | 5527.4 | -2246.9 | 104.1 | -42885.2 | -21466.5 | -312.6 |
|  | (0.111) | (0.355) | (0.706) | (0.073) | (0.514) | (0.800) |
|  |  |  |  |  |  |  |
| Lund University | 4083.7 | -1876.8 | 157.3 | 11948.0 | 41481.7 | 11194.4^*^ |
|  | (0.562) | (0.624) | (0.660) | (0.812) | (0.585) | (0.025) |
|  |  |  |  |  |  |  |
| IVF | -4097.5 | -3746.4 | 145.8 | 27504.0 | 33092.3 | -1482.4 |
|  | (0.261) | (0.221) | (0.381) | (0.423) | (0.304) | (0.420) |
|  |  |  |  |  |  |  |
| Preeclampsia | 13668.7*** | 7899.1* | 26.12 | -7457.8 | 6535.9 | 399.2 |
|  | (0.000) | (0.017) | (0.880) | (0.682) | (0.693) | (0.739) |
|  |  |  |  |  |  |  |
| Caesarean | -5901.0 | -10841.1 | 3314.7*** | 106341.4* | 119203.6^*^ | 3517.6 |
|  | (0.201) | (0.149) | (0.000) | (0.042) | (0.046) | (0.201) |
|  |  |  |  |  |  |  |
| Livebirth | 14742.6* | 32625.9* | -658.1 |  | 0 |  |
|  | (0.011) | (0.011) | (0.130) |  | (.) |  |
|  |  |  |  |  |  |  |
| Died in NNU |  |  |  | -92054.7* |  |  |
|  |  |  |  | (0.015) |  |  |
| Observations | 117 | 100 | 111 | 81 | 72 | 58 |

NNU = Neonatal intensive care unit, IVF = In vitro fertilization. *p*-values in parentheses. ^*^ *p* < 0.05, ^**^ *p* < 0.01, ^***^ *p* < 0.001

**Table S7.** GLM adjusted regression output for costs calculated using 6-week CSRI.

|  | Community | Hospital | Societal |
| --- | --- | --- | --- |
| Extremely preterm | -337.6^*^ | -1137.1 | -407.3 |
|  | (0.040) | (0.159) | (0.943) |
|  |  |  |  |
| Very preterm | -219.4 | -950.6 | -2479.5 |
|  | (0.141) | (0.190) | (0.651) |
|  |  |  |  |
| Late preterm | -122.3 | -1052.3 | 3580.1 |
|  | (0.435) | (0.343) | (0.475) |
|  |  |  |  |
| Term | 0 | 0 | 0 |
|  | (.) | (.) | (.) |
|  |  |  |  |
| Maternal age at enrolment | 8.334 | 27.11 | -46.37 |
|  | (0.322) | (0.381) | (0.921) |
|  |  |  |  |
| White | 31.52 | 12.38 | -920.6 |
|  | (0.747) | (0.953) | (0.698) |
|  |  |  |  |
| Parity | -28.18 | 11.12 | -2166.9 |
|  | (0.492) | (0.857) | (0.382) |
|  |  |  |  |
|  |  |  |  |
| IVF | 60.29 | 559.4 | -2308.9 |
|  | (0.683) | (0.183) | (0.620) |
|  |  |  |  |
| Preeclampsia | 73.91 | 494.7 | 4648.7 |
|  | (0.447) | (0.365) | (0.202) |
|  |  |  |  |
| Caesarean | 338.4^*^ | 1433.4 | 11335.8 |
|  | (0.047) | (0.150) | (0.347) |
|  |  |  |  |
| Livebirth | -424.7^*^ | -1457.2 | -9543.2 |
|  | (0.034) | (0.176) | (0.471) |
| Observations | 69 | 69 | 65 |

NNU = Neonatal intensive care unit, IVF = In vitro fertilisation

*p*-values in parentheses

^*^ *p* < 0.05, ^**^ *p* < 0.01, ^***^ *p* < 0.001
